# P-1315. Activity of Aztreonam-avibactam and Ceftazidime-Avibactam against Enterobacterales and Pseudomonas aeruginosa Causing Infections in Immunosuppressed Patients from United States Medical Centers (2019-2024)

**DOI:** 10.1093/ofid/ofaf695.1503

**Published:** 2026-01-11

**Authors:** Helio SaderMariana Castanheira, Todd Riccobene, John Kimbrough, Rodrigo E Mendes

**Affiliations:** Element, North Liberty, IA; AbbVie Inc, Madison, New Jersey; Element Iowa City (JMI Laboratories), North Liberty, Iowa; Element Iowa City (JMI Laboratories), North Liberty, Iowa

## Abstract

**Background:**

Aztreonam-avibactam (ATM-AVI) was recently approved by the United States (US) Food and Drug Administration (FDA) for the treatment of intra-abdominal infections. ATM-AVI has shown potent activity against multidrug-resistant (MDR) Enterobacterales, including metallo-β-lactamase (MBL) producers. We evaluated the antimicrobial susceptibility of Enterobacterales and *P. aeruginosa* (PSA) of immunosuppressed patients from US medical centers.

**Figure:**
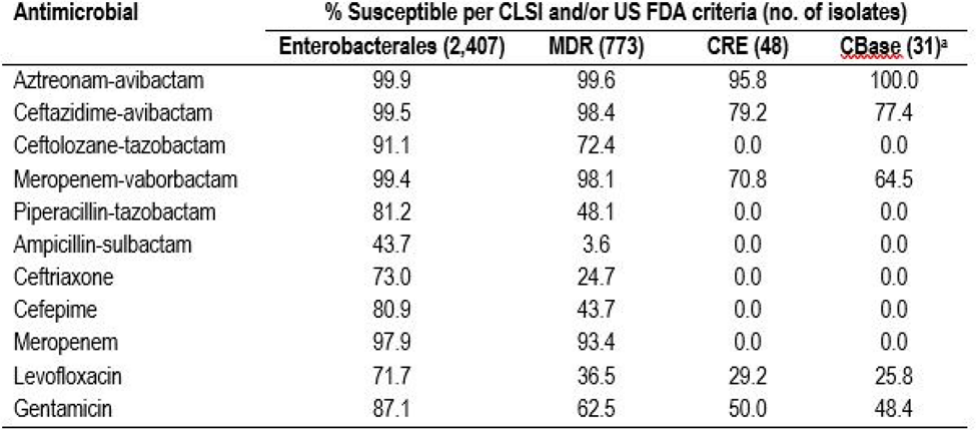
Antimicrobial susceptibility of Enterobacterales and resistant subsets of isolates from immunosuppressed patients a Carbapenemase-producing CRE isolates. Abbreviations: CLSI, Clinical and Laboratory Standards Institute; US FDA, United States Food and Drug Administration; MDR, multidrug-resistant; CRE, carbapenem-resistant Enterobacterales; CBase, carbapenemase.

**Figure:**
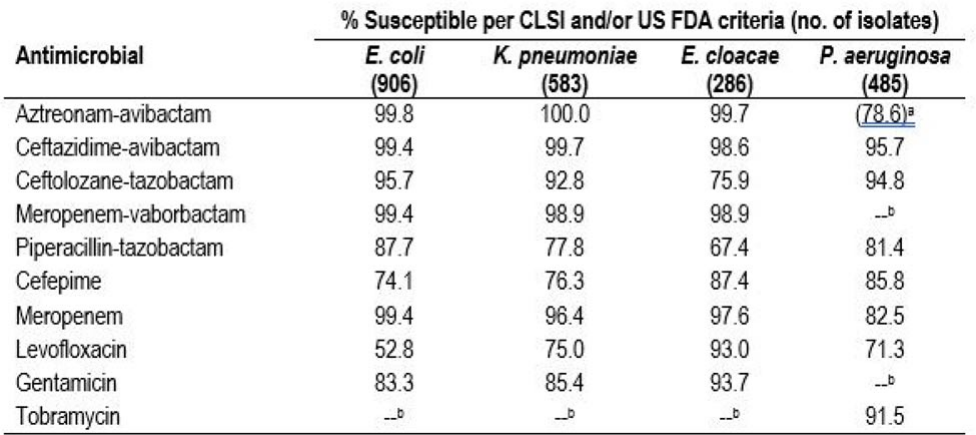
Antimicrobial susceptibility of selected species collected from immunosuppressed patients a % inhibited at ≤8 mg/L, the CLSI breakpoint for aztreonam. b Not tested or no breakpoint published by US FDA. Abbreviations: CLSI, Clinical and Laboratory Standards Institute; US FDA, United States Food and Drug Administration

**Methods:**

Bacterial isolates were consecutively collected (1/patient) from 75 US medical centers in 2019-2024 and susceptibility tested by broth microdilution. Enterobacterales and PSA from patients hospitalized in hematology, oncology, and transplant units were evaluated. Carbapenem-resistant Enterobacterales (CRE; isolates with MIC ≥ 4 mg/L for meropenem and/or imipenem) were screened for β-lactamase by whole genome sequencing.

**Results:**

Enterobacterales were mainly from bloodstream infection (BSI; 53.6%) and urinary tract infection (UTI; 19.9%) and PSA were mainly from BSI (37.9%) and pneumonia (35.0%). ATM-AVI, ceftazidime-avibactam (CAZ-AVI), and meropenem-vaborbactam (MEM-VAB) were highly active against Enterobacterales (99.9-99.4% susceptible [S]), including MDR isolates (99.6-98.1% S; Table 1), ATM-AVI retained potent activity against CRE isolates (95.8% S). Ceftolozane-tazobactam (TOL-TAZ) showed good activity against *E. coli* (95.7% S), and *K. pneumoniae* (92.8% S), but limited activity against *E. cloacae* species complex (75.9% S; Table 2). All (100.0%) carbapenemase (CBase)-producing CRE isolates were ATM-AVI-S while 77.4% were CAZ-AVI-S and 67.7% were MEM-VAB-S. The most common CBases were KPC (61.3%), NDM (16.1%), and OXA-48 types (16.1%). MBL represented 19.4% of CBases. The most active agents against PSA were CAZ-AVI (95.7% S), TOL-TAZ (94.8% S), and tobramycin (91.5% S). PIP-TAZ and meropenem were active against 81.4% and 82.5% of PSA, respectively, and ATM-AVI inhibited 78.6% of PSA at ≤8 mg/L.

**Conclusion:**

ATM-AVI demonstrated almost complete activity (99.9% S) against Enterobacterales, including 100.0% of CBase producers, and both CAZ-AVI and TOL-TAZ were highly active against PSA from immunosuppressed patients.

**Disclosures:**

Helio Sader, United States Food and Drug Administration: FDA Contract Number: 75F40123C00140 Mariana Castanheira, PhD, Melinta Therapeutics: Advisor/Consultant|Melinta Therapeutics: Grant/Research Support Rodrigo E. Mendes, PhD, GSK: Grant/Research Support|Shionogi & Co., Ltd.: Grant/Research Support|United States Food and Drug Administration: FDA Contract Number: 75F40123C00140

